# A Scalp Mass Leading to the Discovery of a Giant Intracranial Arteriovenous Malformation

**DOI:** 10.7759/cureus.80869

**Published:** 2025-03-20

**Authors:** Soufiane Aghbal

**Affiliations:** 1 Radiology, Hôpital Universitaire de Bruxelles (H.U.B) Hôpital Erasme, Brussels, BEL

**Keywords:** arteriovenous malformation, ct angiography, echography, giant avm, intracranial vascular anomaly, magnetic resonance imaging, neurovascular malformation, scalp mass, vascular lesion

## Abstract

Scalp masses are common clinical findings with a broad differential diagnosis, often benign in nature. However, in rare cases, they can be the initial presentation of an underlying intracranial pathology. We report the case of a patient presenting with a painless scalp mass, which led to the incidental discovery of a giant intracranial arteriovenous malformation (AVM). Imaging studies revealed an extensive high-flow vascular lesion with multiple arterial feeders and venous drainage, consistent with a giant AVM. Given the size and complexity of the malformation, a multidisciplinary approach was required for evaluation and management. Giant intracranial AVMs are rare and often diagnosed following neurological symptoms such as seizures, headaches, or hemorrhage. In this case, the scalp mass served as an external marker of a deep-seated vascular anomaly, highlighting the importance of thorough clinical and imaging investigations in atypical presentations. This case emphasizes the need for vigilance when evaluating scalp masses, as they may be the first indication of significant intracranial pathology. Early recognition and appropriate imaging can lead to timely diagnosis and intervention, potentially preventing life-threatening complications.

## Introduction

Arteriovenous malformations (AVMs) are complex vascular anomalies characterized by abnormal direct connections between the arteries and veins, bypassing the capillary network. This results in high-flow arteriovenous shunting, which can lead to vascular steal, venous congestion, and an increased risk of rupture due to hemodynamic stress. AVMs can occur anywhere in the body, but intracranial AVMs are of particular concern due to their potential for life-threatening hemorrhage and neurological impairment. The estimated prevalence of intracranial AVMs in the general population is approximately 0.01%-0.5%, with giant AVMs (>6 cm nidus size) representing a rare but significant subgroup [1,2].

Intracranial AVMs are typically diagnosed following neurological symptoms such as seizures, headaches, or intracranial hemorrhage [2,3]. However, in rare cases, they may present with atypical findings, including extracranial manifestations like scalp masses. The presence of an associated scalp mass is unusual and may indicate a high-flow extracranial component or venous congestion secondary to an extensive intracranial shunting system [4,5]. These high-flow vascular malformations can lead to progressive scalp swelling, pulsatile masses, and, in some cases, skin ulceration or hemorrhage.

In such cases, multimodal imaging is essential for an accurate diagnosis and comprehensive evaluation of the lesion’s extent. Magnetic resonance imaging (MRI), computed tomography angiography (CTA), and Doppler ultrasound play a crucial role in assessing the hemodynamic characteristics and vascular anatomy of the anomaly [6].

We report the case of a patient in whom the incidental discovery of a painless scalp mass led to the diagnosis of a giant intracranial AVM. This case highlights the importance of recognizing atypical presentations of AVMs and underscores the need for a thorough imaging assessment to guide management and prevent potential complications [7,8].

## Case presentation

**Figure 1:**
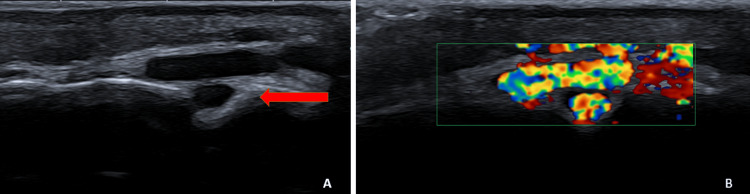
Ultrasound imaging of the scalp mass (A) B-mode ultrasound image showing a vascular mass in the scalp. A possible intracranial extension through a bony defect is suggested (red arrow). (B) Color Doppler ultrasound demonstrating significant vascular flow within the lesion

A 58-year-old asymptomatic male patient presented to the imaging department for the evaluation of scalp swelling. Clinical examination revealed a soft, pulsatile mass in the left parietotemporal region. Initial ultrasound examination demonstrated a vascular structure with a possible intracranial trajectory through a bony defect (Figure 1).

Before imaging, the primary clinical suspicion was a vascular anomaly due to the painless and noninflammatory nature of the scalp mass. Differential diagnoses included a benign vascular tumor (e.g., hemangioma) or a low-flow vascular malformation. However, the progressive enlargement and pulsatile nature of the lesion suggested a high-flow arteriovenous shunting process. Similar cases in the literature highlight the importance of considering AVMs in the differential diagnosis of atypical scalp masses, especially when signs of arteriovenous shunting are present.

**Figure 2:**
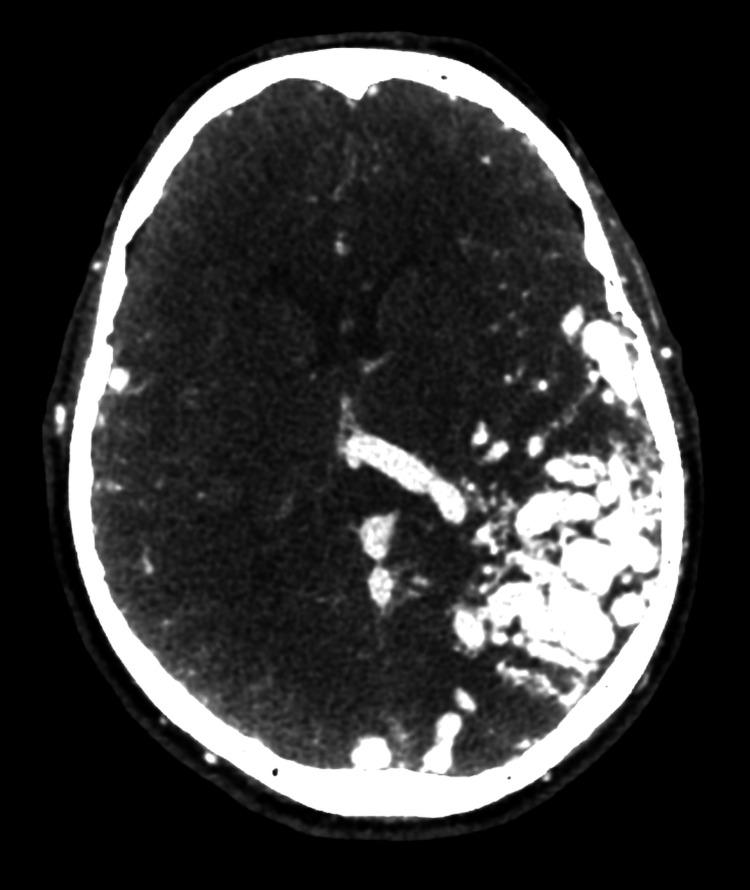
CTA of the giant AVM CT: computed tomography angiography; AVM: arteriovenous malformation Contrast-enhanced CTA revealing a giant left parieto-temporo-occipital AVM

To further assess the vascular anomaly, a CTA was performed. The CTA revealed a giant left parieto-temporo-occipital AVM measuring approximately 6 cm (Figure 2). The AVM was supplied by multiple arterial feeders, mainly originating from the middle cerebral artery and the posterior cerebral artery. Venous drainage was significantly ectatic, involving the superior sagittal sinus, the straight sinus, the vein of Galen, the perimesencephalic venous plexus, and the basal vein of Rosenthal.

**Figure 3:**
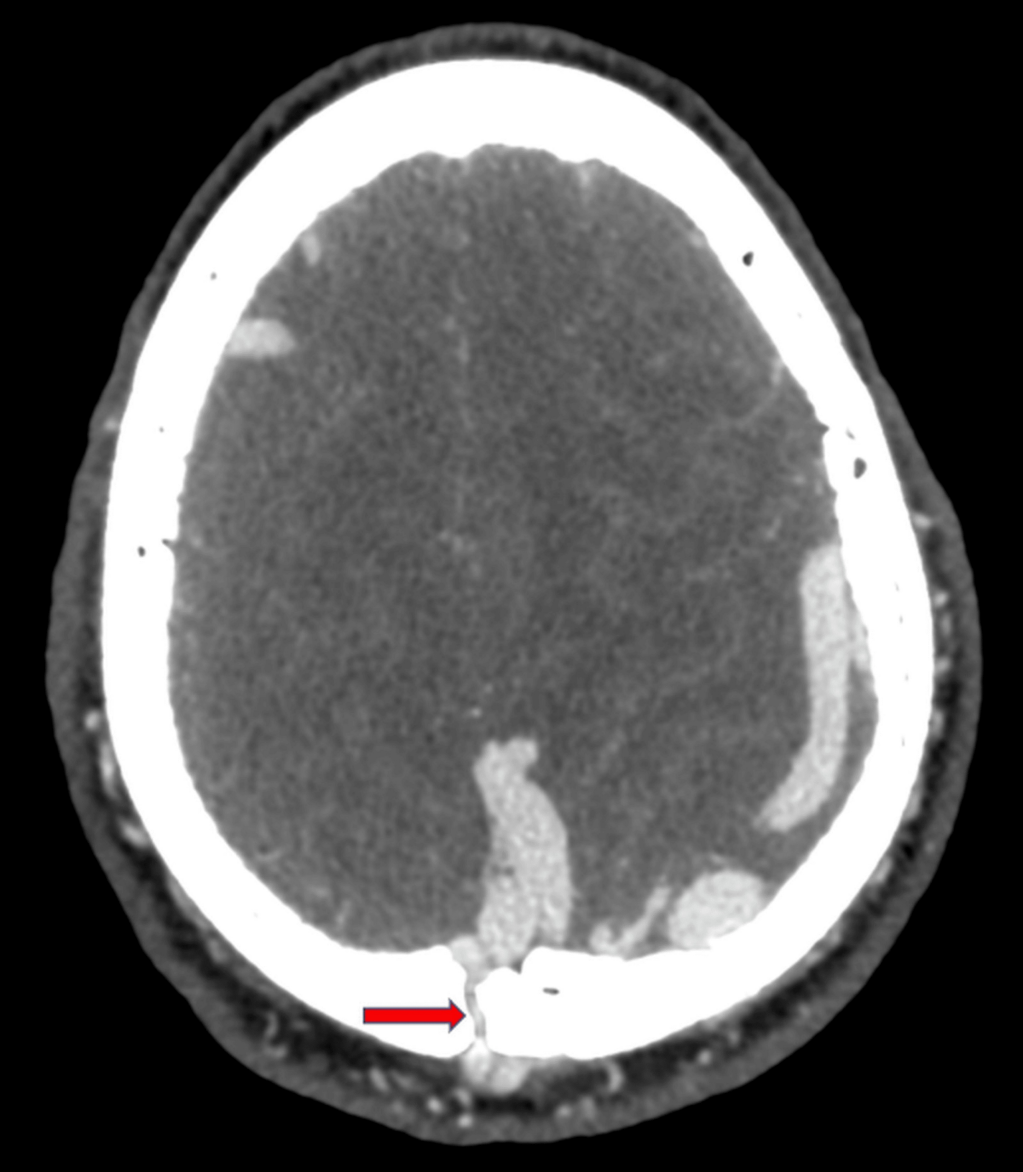
CTA demonstrating a sinus pericranii CT: computed tomography angiography Contrast-enhanced CTA showing a sinus pericranii (red arrow), illustrating the abnormal communication between the superior sagittal sinus and the dilated extracranial vein (corresponding to the scalp mass) via a transosseous emissary vein

The vascular structure initially detected on ultrasound corresponded to a sinus pericranii (Figure 3), an abnormal communication between the intracranial dural sinuses and the extracranial venous structures via an emissary transosseous vein. This rare entity is often associated with high-flow vascular anomalies such as AVMs.

This case illustrates an unusual presentation of a giant intracranial AVM, initially detected as an isolated scalp mass, emphasizing the importance of multimodal imaging in diagnosing atypical vascular lesions. The patient was referred to the interventional neuroradiology department for further management. Given the size and complexity of the AVM, a multidisciplinary discussion was planned to assess the feasibility of an endovascular treatment. The patient is currently awaiting evaluation by specialists in neurointerventional procedures, who will determine the most appropriate therapeutic approach. Regular follow-ups will be conducted to monitor the progression of the lesion and assess potential treatment options.

## Discussion

**Figure 4:**
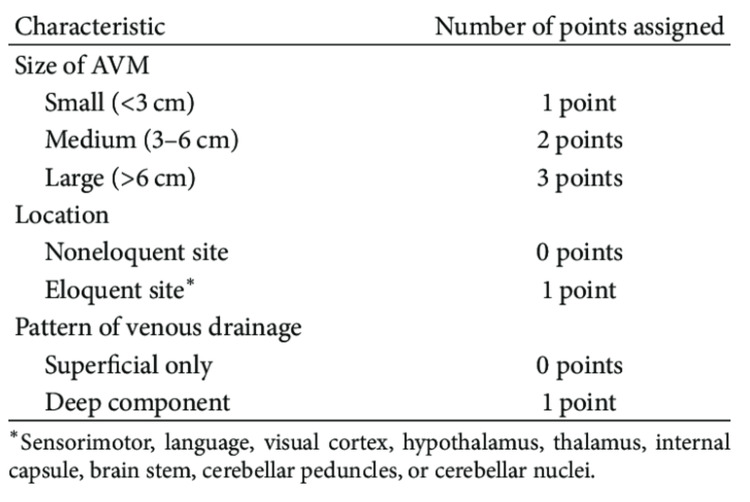
Spetzler-Martin grading scale

Intracranial AVMs are congenital vascular anomalies characterized by direct connections between arteries and veins, bypassing the capillary network. While most AVMs present with neurological symptoms such as seizures, hemorrhage, or headaches [8], some rare cases exhibit atypical manifestations, including extracranial involvement like a scalp mass [9]. This case report highlights an unusual presentation of a giant intracranial AVM, initially detected as a scalp swelling, emphasizing the importance of comprehensive imaging in such scenarios. AVMs are commonly classified using the Spetzler-Martin grading system (Figure 4), which assesses size (small <3 cm, medium 3-6 cm, large >6 cm), the eloquence of adjacent brain areas, and venous drainage. Giant AVMs (>6 cm) represent less than 5% of all AVMs but are associated with a significantly higher risk of hemorrhage (up to 60% lifetime risk) and neurological deficits.

Sinus pericranii and its association with AVMs

A key feature of this case is the sinus pericranii, an abnormal venous communication between the intracranial dural sinuses and extracranial veins through an emissary transosseous vein. This entity is rare and has been associated with high-flow vascular malformations, including AVMs [10]. The recognition of a sinus pericranii is crucial, as it may serve as an external marker for an underlying intracranial vascular pathology. In this patient, color Doppler ultrasound first raised suspicion of a vascular anomaly, which was later confirmed by CTA, revealing a giant AVM with extensive arterial feeders and venous drainage.

Imaging and diagnostic challenges

The diagnostic workup of suspected AVMs requires a multimodal imaging approach. While ultrasound can provide initial clues, CTA, and magnetic resonance angiography are essential for assessing the size, arterial supply, and venous drainage of the lesion. In high-flow vascular anomalies, digital subtraction angiography (DSA) remains the gold standard for treatment planning, as it provides a dynamic evaluation of blood flow patterns and potential therapeutic targets. CTA has a sensitivity of approximately 85%-95% and a specificity of 90%-97% for detecting AVMs, making it a reliable, noninvasive tool. However, DSA remains the gold standard, offering nearly 100% sensitivity and superior spatial resolution for assessing angioarchitecture and treatment planning [9-11].

Management considerations

Giant AVMs pose significant therapeutic challenges due to their high risk of hemorrhage, progressive venous congestion, and difficult surgical access. Management strategies include endovascular embolization, surgical resection, and stereotactic radiosurgery, often requiring a multidisciplinary approach [11]. In this case, the presence of a sinus pericranii further complicates treatment decisions, as its role in venous drainage must be carefully evaluated before any intervention.

## Conclusions

This case underscores the importance of considering intracranial vascular malformations in the differential diagnosis of scalp masses, especially when associated with vascular features on imaging. The presence of a sinus pericranii should prompt further investigations to rule out an underlying AVM. A multimodal imaging approach is essential for accurate diagnosis and optimal treatment planning to prevent complications. This case highlights key diagnostic challenges, reinforces the need for thorough vascular assessment, and emphasizes the importance of interdisciplinary collaboration in guiding management decisions, ultimately contributing to improved patient outcomes.
